# Emergent gambling advertising; a rapid review of marketing content, delivery and structural features

**DOI:** 10.1186/s12889-021-10805-w

**Published:** 2021-04-14

**Authors:** Jamie Torrance, Bev John, James Greville, Marie O’Hanrahan, Nyle Davies, Gareth Roderique-Davies

**Affiliations:** Addictions Research Group, School of Psychology and Therapeutic Studies, University of South Wales, Pontypridd, CF37 1DL UK

**Keywords:** Gambling, Advertising, Marketing, Review, Public health

## Abstract

**Background:**

Gambling advertising is well-funded and has become increasingly sophisticated in recent years. As the presence and pervasiveness of gambling advertising increases, there is a corresponding need for empirical understanding of the characteristics and trends associated with emergent gambling advertisements and marketing. However, there is limited data on this rapidly evolving phenomenon.

**Methods:**

A rapid review was undertaken of the empirical research (2015–2020) that focused upon the content, delivery and structural features incorporated within emerging gambling advertising.

**Results:**

Twenty-five studies were included in the review. The majority of these studies were conducted in either the UK or Australia; two jurisdictions that have unique and particularly liberal gambling environments. The literature suggests that emergent gambling advertising content is targeted, positively framed and in some instances, may overrepresent riskier bets. The sporting and social media spheres are densely populated with such advertisements that involve both direct and indirect marketing strategies. In relation to the online environment, there is evidence to suggest the emergence of more interactive advertisements that prompt user engagement. In addition, financial incentivisation has diversified and is often subject to strict and esoteric conditions. Despite these emergent trends, little provision is devoted to adequately displaying harm reductive or responsible gambling content within gambling advertising.

**Conclusions:**

Overall, there is a paucity of research and lack of methodological diversity concerning the characteristics of advertising within the literature. The barriers to investigating emerging gambling advertising are discussed alongside future research priorities. It is important for this research area to expand in order to appropriately inform ethical industry marketing and effective harm-reduction strategies. *[Pre-registered online* via *Prospero: CRD42020184349].*

**Supplementary Information:**

The online version contains supplementary material available at 10.1186/s12889-021-10805-w.

## Background

The complexity and availability of gambling continues to grow on an international scale [1, 2]. In recent years, there has also been a corresponding increase in the prevalence, diversity and intensity of gambling advertising [3, 4]. This expansion is facilitated by significant industry expenditure; especially within jurisdictions that have previously liberalised gambling such as the United Kingdom (UK) and Australia. Estimates indicate that Australian gambling industry spending on marketing and promotion has increased by 33% per year since 2011 to $273 million in 2018 [5]. UK industry spending grew over 17% per year from 2014 to 2018, reaching an estimated total of £1.5 billion [6]. This advertising expenditure represents 10.34% of the £14.5 billion gross yield of the UK gambling industry in 2018 [7]. Such funding has led to the development of sophisticated advertising campaigns that are disseminated across traditional media such as television [8] and via sports sponsorship [4]. In addition, these campaigns have resourcefully adapted to the digital sphere via online and social media marketing [9, 10]. This shift towards the online environment has granted gambling operators uninterrupted advertising space; especially during the Covid-19 pandemic. Therefore, attempts to curtail TV gambling advertising (as seen within the UK) during periods of lockdown may have little effect on reducing overall exposure amongst young or vulnerable audiences [11].

Emerging literature has highlighted gambling as a compounding issue of public health [12, 13]. The harmful effects of gambling and associated advertising have been suggested to extend beyond populations of disordered gamblers and are apparent across the entire harm-spectrum; including children and young people [3, 14, 15]. Comparable to previously conducted reviews of alcohol and tobacco [16, 17], two recently published systematic reviews [18, 19] and one narrative review [20] have indicated that gambling advertising is facilitative of induced gambling intentions or cravings, increased participation and riskier (more impulsive) betting. However, these reviews also identify many of the methodological gaps within the existing gambling advertising research. Within the literature there is an emphasis placed upon the self-reported effects of gambling advertising exposure, especially amongst disordered gamblers. An empirical concentration upon disordered gamblers may pathologize the issue of gambling-harm induced by advertising. This may draw attention away from advertising-induced harm experienced by low-moderate risk gamblers [18]. Furthermore, the self-reported effects of gambling advertising are often hindered by recall and self-report bias. This may be due (in-part) to the Third Person Effect [21, 22] in which individuals are more likely to perceive the impacts of marketing amongst others rather than themselves. In contrast, there is a paucity of research that focuses upon the specific characteristics and mechanisms that underpin emergent gambling advertisements.

There is a growing academic consensus that gambling advertising may incorporate content that is deemed misleading, utilises demographic targeting and uses embedded promotion [22–24]. However, to date, no review has aimed to provide a taxonomy of gambling advertising characteristics. As observed in the movement towards increased control of tobacco advertising [25–27], studies that aim to investigate the specific marketing methods utilised by the industry offer an insightful contribution in the shift towards regulatory reform and industry marketing that is more ethical and transparent. Therefore, the current review of gambling advertising characteristics seeks to complement the existing reviews of advertising effect as well as the future literature. This contribution is also warranted in order to appropriately inform the decisions of policymakers and researchers regarding effective harm-reduction strategies.

Due to the fluctuating methods of gambling advertising that largely remain free from effective regulation [28], this review aimed to examine the empirical evidence concerning the nature and characteristics of emerging (2015–2020) gambling advertisements. Specifically, this review aimed to investigate:

- The content and narratives incorporated within gambling advertising.
- The methods of gambling advertising delivery and placement.
- The mechanics and structural features of gambling advertising e.g. design, usability and complexity.

## Methodology

Due to the fluidity and constant development of the gambling advertising sphere, a rapid review methodology was utilised throughout the literature search. Although there is no single accepted approach, the rapid-review process typically involves the same components as a systematic literature review with limitations imposed on the length (e.g. time spent) and depth (e.g. extent of searching) of the methodology [29]. Despite the variation in approaches, rapid reviews have been reported to produce equivalent findings to systematic reviews if screening, bias/quality appraisal and data synthesis are addressed with appropriate methodological rigor [30, 31]. The protocol for the current review was registered via Prospero (*ID: CRD42020184349*).

### Search strategy

**Fig. 1:**
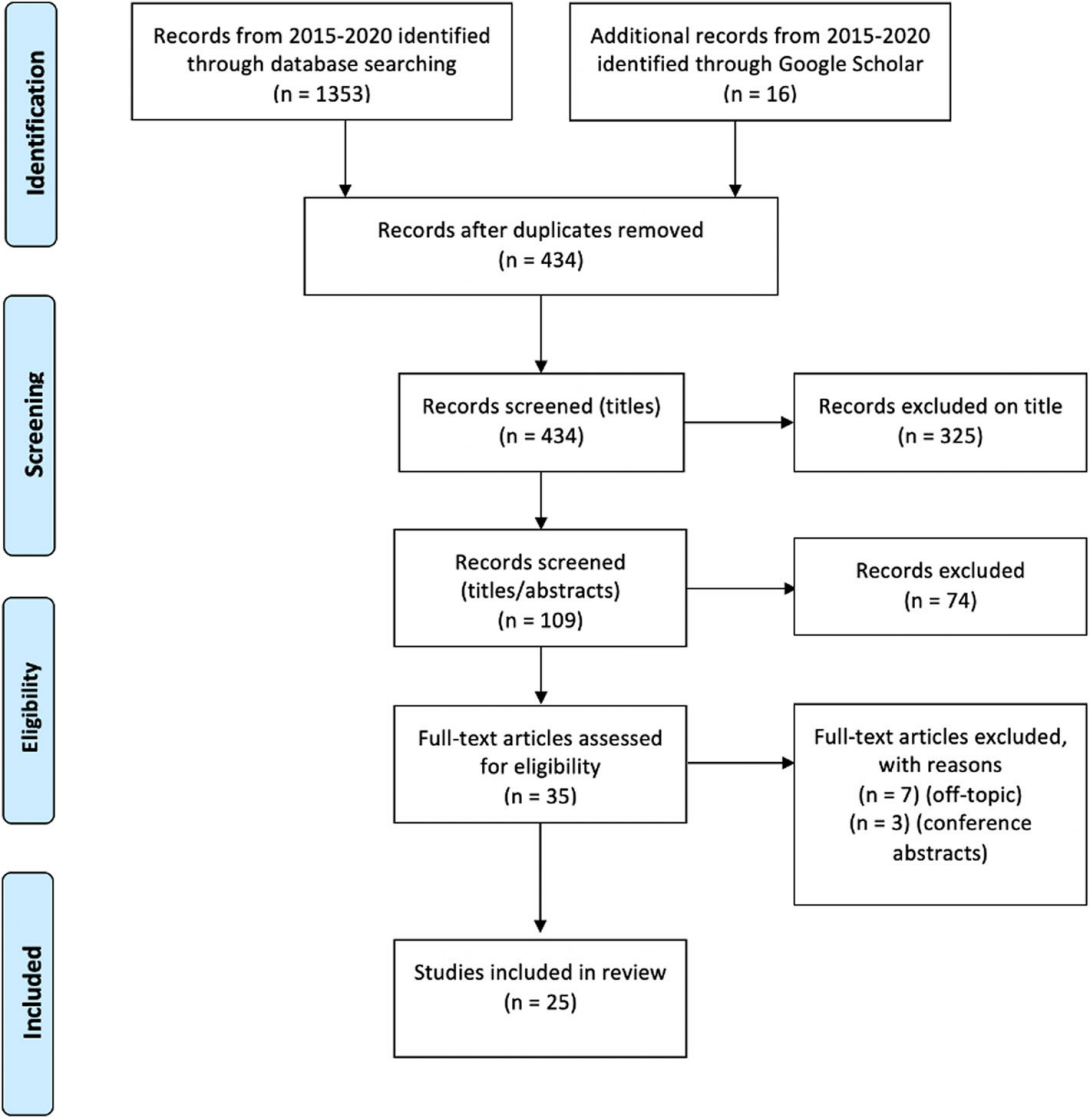
Preferred Reporting Items for Systematic Reviews and Meta-Analyses (PRISMA) flow diagram [32]

Following PRISMA guidelines [32], a literature search for peer-reviewed articles published since 2015 (completed June 2020) exploring the content, delivery and characteristics of emergent gambling advertising was conducted (Fig. 1). Within the search strategy, operational definitions were created for the terms “advertising”, “marketing” and “promotion”. Advertising was defined as any industry financed communication that utilises varying media sources (such as TV or internet ad space) to encourage engagement with a gambling brand or product. Marketing and promotion were operationalised interchangeably and were defined by broader strategies that aim to encourage gambling brand awareness or indirectly influence user engagement (such as sporting sponsorship or affiliate marketing). Therefore, non-industry funded sharing of gambling-related material (such as the independent social media posts of bettors) were not included in the current search strategy. Two academic literature databases were utilised during the search strategy including PsycInfo (via Proquest) and Web of Science (Science Citation Index Expanded & Social Sciences Citation Index). A further set of records were also accrued using Google Scholar. Boolean operators (AND/OR) were used interchangeably during the search strategy in conjunction with the following terms: gambl*, bet*, casino, sport*, market*, advert*, promot*, content* and strateg*. The details of this search strategy can be found in the Search Strategy Report (see Additional file 1). An inclusive approach was undertaken given the general paucity of literature in this field alongside the heterogeneity of the methodologies across emergent studies. Due this heterogeneity, a meta-analysis was not conducted.

### Inclusion/exclusion criteria

Empirical studies (quantitative, qualitative and mixed methods) in the English language were included within the rapid review if they addressed the components, designs, incorporated mechanisms and/or delivery of gambling advertising or marketing. The search was conducted in English as the translation of non-English language articles was unfeasible due to time and economic constraints. Eligible studies were also required to have been published between 01/01/2015 and 02/06/2020. This timeframe was implemented due to the current review focusing upon the characteristics of emergent or recent gambling advertising given how rapidly advertising trends shift and fluctuate. Due to the typical limitations that are placed on the length (time spent) of the rapid review methodology, a practical limit of 5 years was therefore placed on the inclusion criteria. All samples of advertising were eligible for inclusion in order to provide a broad range of synthesised narrative findings. Records were excluded if they were published prior to 2015, were discussion or commentary articles, were not published in the English language, or focused primarily on the self-reported effects of and/or perceptions towards gambling advertising.

### Screening and quality assessment

Following the retrieval of records via database searching (*n* = 1353) and Google Scholar (*n* = 16), duplicates were removed, and an initial title screening process was undertaken (*n* = 434) in order to exclude records that were irrelevant or not applicable. The remaining record title and abstracts (*n* = 109) were screened by three reviewers (JT, MOH and ND). To ensure fidelity during this process, the reviewers regularly met to discuss their individual decisions and reasoning behind including or excluding records until consensus was reached. Following this, full-text screening of 35 records took place against the inclusion/exclusion criteria, with consultations carried out among the wider research team. Any disagreements were also addressed by this team until a general consensus had been attained. The research team included (but was not limited to) three senior researchers with experience in both the subject matter and the review process. Full-text screening led to the exclusion of ten records due to them being off-topic (*n* = 7) or conference abstracts (*n* = 3). A final set of empirical records (*n* = 25) underwent quality assessment via the Mixed Methods Appraisal Tool (MMAT) 2018 [33]. The research team determined that all of the final empirical records were conducted to a good methodological standard according to the MMAT and were subsequently included for full data extraction.

### Analysis/synthesis

In order to distinguish appropriate and salient themes within the included articles, narrative synthesis was conducted. This process involved repeated readings of the literature, extracting relevant content, and summarizing this content in tabular format (see Additional file 2). This information was then synthesised and organised in order to produce a thematic framework. This framework was used to structure the findings according to themes in line with the research aims of the current review. Narrative synthesis was conducted by JT with regular consultation among the co-authors to ensure the applicability and pertinence of the final themes.

## Findings

Twenty-five studies were included in the review: 6 qualitative studies; 15 that employed a mixed-methods approach and 4 quantitative studies (see Additional file 2). The studies were conducted across four jurisdictions that included the United Kingdom (*n* = 12), Australia (*n* = 9), Sweden (*n* = 1) and cross-culturally between the United Kingdom and Spain simultaneously (*n* = 3). The included studies were categorised across three overarching themes (Table 1) in line with the research aims of the current review: 1) Content and narratives 2) Delivery and placement; 3) Structural features and mechanics.

**Table 1:** Summary of themes that emerged as a result of narrative synthesis

Overarching theme	Sub-theme
Content and narratives	• Targeted content that positively frames gambling
	• Odds-related content and promoting complex bets
	• Financially incentivising content
	• ‘Responsible gambling’ and harm-reductive content
Delivery and placement	• The expansive placement of gambling advertising in and around sports
	• Disseminating promotional gambling content via social media platforms
Structural features and mechanics	• Utilising digitally interactive features for marketing purposes
	• Conditions and requirements of advertised bets and offers

### Content and narratives

The content and narratives that are incorporated into gambling advertising were outlined in 23 studies. Four sub-themes emerged that included: 1) Targeted content that positively frames gambling; 2) Odds-related content and promoting complex bets; 3) Financially Incentivising content; 4) ‘Responsible gambling’ and harm-reductive content.

#### Targeted content that positively frames gambling

A number of content analyses highlighted the themes and latent messages that were utilised across a range of gambling advertisements that portray gambling as a desirable, trustworthy and fun activity. In relation to casino gambling, a study of UK online casino marketing identified the use of language within advertisements that predominantly orientated positive emotions such as trust and joy [34]. In-venue casino advertisements were also positively framed. For example, Australian social club (casino) endorsements were found to portray the venues as being accommodating, comfortable and well-equipped [35]. These advertisements often aimed to emphasise better value for money and attempted to accentuate an increased chance of success in relation to the gambling activities they offered. Potential customers were encouraged to bring their family (including children) to such establishments due to the availability of non-gambling related amenities provided inside [35]. This positive framing was also observed within online social casino (free-to-play gambling) advertisements in which bright colour schemes and themes associated with glamour and financial success were employed [36]. Such advertisements were deemed likely to appeal to young people due to the incorporation of cartoon animal characters and novel pop-culture references [36]. Additional studies of wagering advertising supported this finding by citing the use of content that contained animations, memes, humour and celebrity endorsement within advertisements that may have particular appeal to children and young people [37, 38].

In relation to gendered content, one Swedish study of TV marketing indicated that female casino gamblers were visually overrepresented within potentially targeted advertising content compared to males [8]. Female-orientated content was also observed in relation to the marketing of UK-based bingo websites [39]. Such websites included the use of ‘feminine’ colour schemes alongside light-hearted, fun and reassuring content that aimed to create a sense of belonging for new customers. Bingo was also predominantly portrayed as a benign activity to engage with regularly [39].

Contrastingly, numerous studies of sports betting advertising highlighted the male-orientated focus of incorporated content [40–42]. For example, Australian operators positively framed sports betting via themes such as thrill, peer bonding, power/control and sports-fan rituals [40]. This trend was also observable across other jurisdictions such as the UK and Spain in which televised football betting advertisements were male-dominated and visually combined gambling participation, drinking alcohol and emotionally charged situations such as celebrating a goal and peer bonding [41, 43]. A further study conducted by Lopez-Gonzales et al. [42] re-examined these British and Spanish advertisements in terms of their conceptual metaphors and concluded that operators aimed to align love for a team with betting on that team and portrayed sports betting as a rational market in which the smart succeed. In addition, the positive framing of sports betting within UK advertisements may also be facilitated via the use of a dual-persuasive strategy that aims to reduce perceived risks whilst increasing perceptions of increased control. This persuasive content strategy was highlighted in one study that distinguished the incorporation of positive themes that oriented around ‘free’ money and fun whilst emphasising the advantageous effects of knowledge and sports-related data analysis within a masculine context [44].

From a broader perspective, the current review revealed a less overt positive framing of gambling by operators who utilised social media to build brand awareness amongst audiences and form positive relationships with customers. Studies conducted in both the UK and Australia highlighted the online posting of less commercial content by operators. This included posting related news and upcoming events as a means of positively normalising gambling within a broader social context [45, 46]. In a qualitative interview study of Australian gambling industry employees, participants disclosed sharing stories of customer wins and posting interesting news content with the aim of targeting specific audiences; sometimes this audience included young adults, while on other occasions content was directed towards higher profile social media users for the sake of brand exposure [47].

#### Odds-related content and promoting complex bets

Several studies highlighted the dissemination of specific odds or betting-related information and content by operators within the context of sports betting. One Australian study identified that the indirect or non-commercialised approach utilised on social media platforms used to build brand awareness was often interwoven with specific odds-related content with the aim of keeping customers informed [47]. Other studies focused upon the betting and odds-related content disseminated via televised sports betting advertisements. For example, in an investigation of UK and Spanish advertising depictions of betting behaviour, it was determined via qualitative content analysis that bettors were frequently shown to be partaking in ‘in-play’ betting via the use of smart-phones and laptops [43]. This emergent form of betting refers to the placement of wagers on an ongoing event that is yet to finish; bets can be modified by the user as the event progresses meaning they are often more complex and have longer odds compared to more conventional forms of sports betting [43].

The current review revealed a skewed representation towards such complex bets as well as other ‘exotic’, ‘special’ or high stakes wagers within UK televised football betting advertisements. Specifically, such advertisements were more likely to depict and promote these complex bets in comparison to more simple bets during matches throughout the English Premier League [4] and during the 2018 World Cup [48, 49]. The authors argued that this was facilitated via a qualitative trend amongst the advertisements that is theoretically designed to nudge bettors through multiple channels towards more impulsive and high-risk bets with larger potential payoffs [4, 48, 49]. This was also observable amongst conventional gambling advertisements within UK bookmaker shop windows during the 2014 World Cup [50]. It was found that odds-related content associated with complex bets was advertised almost exclusively via this method [50]. No included studies focused on comparing the depiction of complex vs simple sporting bets within other jurisdictions such as Australia or Sweden. It appears this topic has most thoroughly been investigated in the UK thus far. However, it is acknowledged that studies published in languages other than English may have also examined this topic but were subsequently excluded from the current review during the literature search.

#### Financially incentivising content

The current review identified a prominent theme of operators incorporating financial incentives into advertising content that took a wide range of forms. Within the included studies financial incentives were characterised by their intended purpose of encouraging gambling amongst consumers by providing them with inducements, offers and promotional deals such as ‘free bets’, bonuses and matched deposits [51]. In comparison to traditional media sources such as television, financial incentives are often disseminated digitally via mobile and social media sources that do not typically adhere to established advertising restrictions [51]. Although financial incentives are distributed within the context of various gambling types [8, 39], they are most commonly associated with sports betting [45, 51–53].

The extensive variability of gambling-related inducements and offers was highlighted within an Australian study that identified 15 different types of incentivising content [51]. This included; sign-up offers, refer a friend offers, happy hours, refund (stake back) offers, odds-bonuses and winnings paid back to the consumer despite an unsuccessful bet [51]. Such content was often disseminated by Australian sports betting operators via social media [38, 46], direct emails, and texts [53]. Similarly, UK gambling operators often included inducement and offer-related content within their Twitter posting [45, 52] as well as televised gambling advertisements within a sports betting context [44]. To a lesser degree, televised Swedish casino advertising [8] and UK-based bingo websites [39] were also identified for their use of financially incentivising content aimed at prompting customer engagement.

#### ‘Responsible gambling’ and harm-reductive content

Several studies identified a significant lack of ‘responsible gambling’ (RG) and harm-reduction messaging within the advertisements disseminated by gambling operators across a range of formats. This type of messaging typically takes the form of age restriction information, terms and conditions (T&Cs), signposting towards support services and warnings of the negative consequences of gambling [54]. The included studies focused upon such content assimilated into or presented alongside the marketing or promotion of gambling brands, products and offers. Investigations of standalone harm-reduction or RG campaigns that fall outside of the commercial advertising efforts of the industry were not included.

In a study of Australian social casino advertisements distributed via social media, it was determined that little provision was given to such messaging in which nearly 90% of all analysed adverts (*n* = 115) contained no content aimed to protect consumers from gambling-related harm [36]. Similarly, this lack of harm-reductive messaging was also observed amongst other social media advertisements for Australian casino venues, lottery venues, electric gaming machine (EGM) venues and sports betting operators [46]. Individual inducements and offers on Australian wagering websites were also highlighted for their significant lack of RG messaging alongside lengthy T&Cs that often incorporated complicated legalistic language [51]. Although 95% of the analysed websites (*n* = 223) displayed some form of RG message on the home page, they were characterised by their lack of prominence and visibility [51].

UK-based studies of gambling advertising produced comparable findings in which Twitter posts from operators and affiliates (third parties) contained very few RG and harm-reduction messages [45, 52]. In relation to UK televised sporting events, one study highlighted that only 1% of visual and verbal promotional gambling advertising references within boxing and 3% in football contained age restriction or harm-reduction messaging [55]. Correspondingly, a comprehensive analysis of printed, radio, internet and televised gambling advertising in the UK (*n* = 300) found that one in seven adverts did not feature age restriction or harm reduction messages whilst one in ten did not contain T&Cs [54]. Within adverts that did contain this content, such messages and information were characterised by very poor visibility and were unlikely to be displayed within the main frame of the advert. The majority of harm-reduction messages within the analysed advertisements failed to explicitly mention gambling-related harm [54].

### Delivery and placement

The emergent delivery and placement of gambling advertising was outlined within 15 studies. Two sub-themes emerged that included: 1) The expansive placement of gambling advertising in and around sports; 2) Disseminating promotional gambling content via social media platforms.

#### The expansive placement of gambling advertising in and around sports

The reviewed studies primarily focused upon the more emergent developments between gambling advertising and televised sports over the past 5 years [4, 48–50, 55]. Only one study retrospectively assessed the prevalence of gambling within sports over the previous two decades. This was conducted via an investigation that tracked the frequency of gambling-related shirt sponsorship within English and Scottish Premier League football matches between 1992 and 2018 [56]. The authors concluded that over the measured period, the gambling industry had significantly increased the frequency of gambling-related shirt sponsorship; especially within the English Premier League. The beginning of this rapid increase coincided with the introduction of the Gambling Act of 2005 in which UK gambling rules and regulations were liberalised [56].

The prominence of gambling advertising broadcasted around UK televised football was also highlighted in other studies that investigated the presence of commercial-break gambling advertisements that aired during 2016 Premier League matches and the 2018 World Cup [4, 48, 49]. During the 2018 World Cup, 69 televised ‘live odds’ advertisements were shown across 32 matches by five bookmakers on British television [49]. In comparison, 63 instances of ‘live odds’ betting were depicted within televised gambling advertisements across 2 months (28 matches) of 2016 Premier League football matches [4]. It should be noted that these analyses focused specifically upon ‘live odds’ advertisements and did not include the other forms of televised football betting advertisements that also aired during this period [4, 49].

Due to such high levels of commercial-break advertising, UK gambling operators agreed to a voluntary ‘whistle-to-whistle’ ban on such promotions before 21:00 in 2019 [55]. However, in an investigation of embedded (within play) gambling advertising that falls outside of the ‘whistle-to-whistle’ criteria, significant numbers of visual and verbal promotional gambling references were found in televised football and boxing [55]. A total of 358 promotional gambling references were recorded over one boxing match with an average of 4.70 references per broadcast minute; 2595 promotional gambling references were recorded over five football matches with an average of 2.75 references per broadcast minute. In boxing, gambling-references were most frequently displayed within the ring, whilst in football they were most frequently displayed around the pitch [55].

#### Disseminating promotional gambling content via social media platforms

In congruence with the increasing prevalence and evolution of social media, numerous studies have highlighted the various delivery and placement methods employed via digital platforms to increase the exposure of gambling advertisements amongst online audiences [34, 36, 38, 45–47, 52]. Traditional media sources such as television and printed media are still being utilised by the gambling industry to promote products [8, 50, 54]. However, the global reach of social media platforms may provide operators the opportunity to significantly increase brand awareness, attract new customers and provide efficient customer relationship management [45].

The platforms used by operators and affiliates to post gambling advertising and promotions included Facebook [36, 38, 46, 47], YouTube [38] and most notably Twitter [34, 38, 45–47, 52]. An Australian interview study of gambling industry employees found that these social media platforms were utilised for specific purposes; Facebook was used primarily for providing rapid feedback to customer queries whilst Twitter was predominantly used for broadcasting gambling-related news and information [47]. Interviewees also stated that it was common practice to pay for targeted advertising space on social media rather than utilising the broader approach of blanket advertising [47].

It has been previously noted that sports betting operators and affiliates maintain a prominent online social media presence for promotional and marketing purposes [46]. Three studies in the current review focused specifically upon the marketing activity and delivery methods of gambling operators and affiliates on Twitter [34, 45, 52]. The authors highlighted the potentially high volume of promotional tweets that were posted on a daily basis. In relation to large gambling operators, two studies concluded daily tweeting frequencies ranging between 89 and 202 tweets [34] and 33–398 tweets [52]. Tweets were found to be distributed at peak times during the day and more often on specific days of the week; possibly in synchrony with particular sporting events [34]. Affiliates were shown to tweet more often with an average of 594 tweets per day [45]. Affiliate marketing involves promotion by third-parties who are financed by gambling operators to direct customers towards particular offers or gambling products. This growing technique is mostly utilised via social media in which seemingly independent ‘influencers’ or ‘tipsters’ provide betting suggestions and recommendations [45].

### Structural features and mechanics

The structural features and mechanics that are incorporated into emergent gambling advertising were outlined in 11 studies. Structural features were characterised by the utilisation of design elements or properties that determine how the advertisements are engaged with by users. Mechanics were characterised by the rules, procedures and specifications associated with game types or particular bets. Two sub-themes emerged that included: 1) Utilising digitally interactive methods for marketing purposes; 2) Specific conditions and requirements of advertised bets.

#### Utilising digitally interactive features for marketing purposes

Emergent gambling advertisements have begun to utilise digital features that require user engagement in order to interact with the advertisement, respond to it or share it [34, 36, 38, 45, 47, 48, 52, 53]. These methods are often facilitated by the functionalities provided by social media. For example, Facebook advertisements for social casino games often utilise the ‘activity’ button within their posts [36]. This interactive feature allows the user to directly download the social casino app or automatically opens the web-browser interface of the game [36]. A similar characteristic was also highlighted within the promotional tweets, direct emails and texts from UK and Australian gambling operators in which direct URL links to the associated betting websites were often embedded within the promotional messages sent to consumers [34, 53].

Two Australian studies also distinguished gambling advertisements that encouraged user-interaction via social media [46, 47]. Audiences were often prompted to use the ‘comment’, ‘like’ and ‘share’ functions in relation to operator posts for the sake of brand-exposure [46, 47]. Another strategy of increasing brand-exposure involved the utilisation of specific Twitter hashtags that reference particular sporting events or promote certain bets [34, 38, 52]. By doing so, sports betting operators could embed their promotional tweets into popular or trending threads relating to upcoming sporting events that were otherwise non-gambling related [38].

Alongside brand-exposure, hashtag functionality was also offered to potential customers by UK gambling operators as a means of increasing user-engagement [48]. Users could take advantage of hashtags such as ‘#getaprice’ and ‘#yourodds’ that allow them to create their own bets by requesting odds for combined events (complex bets) of their choice. The gambling operator then replies back to the user with the odds for their requested bet [48]. This interchange was also commonly performed on a more personal level in which Twitter users could send public or direct messages to operators regarding their customer queries about specific bets, odds and other gambling-related information [34, 45, 52]. Less overt interactions were also observable via Twitter in which UK operators aimed to increase customer engagement by utilising the ability to embed online-polls into their tweets [45, 52]. These polls often posed seemingly innocuous sports-related questions to users in which the promotional intent of the post is not made explicit [45, 52]. Examples of such polls include ‘*Will Harry Maguire score against Manchester United?’* (posted by Bet365 in 2018: [52] and ‘*What’s been the best goal of the World Cup so far*?’ (posted by SkyBet in 2018: [45]. Although the use of digitally interactive features of marketing was evident across numerous gambling formats [36, 47], the evidence suggests they were overwhelmingly utilised within a sports betting context. This is likely due to the incorporation of live (sports-related) information and high level of customisation observable within sports betting. Currently, such elements appear to drive operator use of interactive features and therefore prompt interactive engagement amongst audiences more than other forms of gambling.

#### Conditions and requirements of advertised bets and offers

The mechanics involved with advertised bets and offers were highlighted in two studies that focused upon sports betting in both the UK [49] and Australia [51]. It was determined that many advertised sports betting incentives and inducements had specific conditions, stipulations and play-through requirements that restrict when tangible winnings can be withdrawn from a betting account. These conditions were highlighted for their abstruse and complex nature [51]. For example, a particular sign-up incentive highlighted by Hing et al. [51] offered bettors a 100% matched bonus up to $200 on the condition that they deposited $20 upon opening a new betting account. The conditions also stipulated that bettors needed to stake the deposit amount combined with the amount equivalent to the bonus bet at odds of 1.5 or greater. Bettors were required to do this three times over 3 months. As indicated by Hing et al. [51], “*These play-through requirements meant that it would cost bettors $1000 of their own money for a chance to win from a $200 bonus bet*” (p. 11). Similarly, ‘live-odds’ advertisements disseminated by UK bookmakers have also been shown to possess specific conditions [49]. During the 2018 football World Cup many ‘live-odds’ bets were advertised that were limited in terms of both time and quantity. Furthermore, bets were sometimes shown to be ‘improving’ in odds. The authors suggested that these mechanics may have been strategically designed to make ‘live-odds’ bets appear more urgent than necessary [49].

## Discussion

This rapid review aimed to contribute to the international literature by improving understanding of emergent gambling advertising content, delivery methods and structural features. The evidence suggests that overall, gambling advertising has increased in both complexity and interactivity. In relation to content, previous reviews have highlighted advertising that positively frames or glamorises gambling in a broad sense [57, 58]. However, the current review suggests that this positively framed content has evolved and diversified beyond general glamorisation. This development is especially prominent within male-orientated sports betting advertisements that align gambling with emotionally charged situations, team loyalty and peer bonding [40, 42]. The evidence suggests there may also be an additional form of positive framing within this content that represents themes of increased control whilst underrepresenting themes of risk via a dual persuasive strategy [44]. Positively framed advertising content may also be orientated towards young adults [36], parents [35] and women [39]; although further research is warranted with regards to these groups.

The pattern of results also points towards the depiction and promotion of complex, in-play and exotic bets compared to simple bets within the content of UK football betting advertisements. There may be an economic underpinning to this marketing technique as complex bets are subject to longer odds, equating to potentially higher profit margins for the gambling industry [49]. In addition, such bets may facilitate the emergent transformation of sports betting into an accelerated, continuous and more impulse-driven form of gambling [49, 59]. The current review also suggests that the dissemination of incentivising gambling content such as inducements and offers continue to remain prominent methods of encouraging potential customer engagement. These incentives now take many forms [51], are increasingly complicated, and are pervasively advertised [44, 52]. Contrastingly, much less provision is given to content that contains RG or harm-reductive messaging within gambling advertisements. The included studies indicated that such content is inconsistent, characterised by low visibility and sometimes completely absent [46, 54]. In their current form, such messages have been highlighted for their likely inadequacy in reducing gambling-related harm. For example, a recent eye-tracking study of bettors and non-bettors demonstrated that very few visual fixations are placed on these messages in comparison to other wagering information displayed within sports betting advertisements [60]. Moreover, when specific RG messages are in fact actively perceived by bettors, the messages may fail in terms of their supposed purpose. An example of such message includes the popular UK RG slogan ‘when the fun stops, stop!”. This specific message was identified in approximately two-fifths of the advertising sample utilised by Critchlow et al. [54]. A recent study of 3000 gamblers, indicated that this particular message either showed no beneficial effect of curtailing gambling behaviour or produced a backfire effect that influenced increased betting participation [61].

Within UK sports in particular, the placement and delivery of gambling advertising has intensified over the previous 15 years. Sports betting promotions now extend beyond conventional methods of commercial break advertising and into the area of play [56]. Consequently, shirt sponsorship [56], verbal references made by commentators and embedded (ring/pitch side) advertisements [55] are now saturated with gambling-related stimuli. This is likely due to the unique and liberal nature of the 2005 UK Gambling Act. Although this legislation is set for review [62], it is unlikely that gambling-related sponsorship will be completely prohibited within UK sports. However, there is a political and academic consensus that the UK should follow nations like Spain in which gambling sponsorship within football has been prohibited by law [63]. Future research should seek to investigate the emergent placement of gambling advertising within sports across jurisdictions other than the UK that are set to liberalise sports betting such as North America. In the context of the UK, further research is warranted to investigate the online areas into which gambling advertising may be diverted in response to increasingly restrictive and more effective legislation [55, 64]. This transition has already commenced to a certain extent, as evidenced by the increasing presence of gambling advertising across social media platforms [46, 52]. The regulation of advertising across social media is likely to prove difficult given the direct and indirect promotion of gambling within these online spaces. For example, the findings of the current review indicate an emerging trend in which operators utilise seemingly innocuous content to build brand awareness [46] and finance affiliate promotion to implicitly market gambling online [45]. The promotional intent of these methods is not often made explicit. Furthermore, affiliate marketing has been recently questioned in terms of its transparency, sincerity and true benefit to consumers [10]. Due to this increased use of third-parties, affiliate marketing may also operate as a buffer that shifts or obscures the social responsibility of the gambling industry [45].

From a structural perspective, conventional means of disseminating gambling advertising such as television, radio and billboards have necessarily adopted a linear approach in which advertising is a one-way process of stimuli exposure with minimal user-interaction. By comparison, the recent evidence indicates that emergent gambling advertisements have begun to utilise digitally interactive features that provide the opportunity for a more collaborative interchange between the operator and the public [34, 45, 53]. Therefore, the current review recommends the empirical study of the mechanisms and impacts associated with these emergent structural features as a future research priority. This includes promotional URL links sent directly to bettors, gambling-related ‘polls’ posted by operator social media accounts and gambling-related hashtags utilised by consumers.

In relation to the completeness and applicability of these findings, it appears the available evidence is sufficient but not comprehensive in addressing the present research aims. As seen within the sphere of tobacco and alcohol marketing, internal information concerning gambling industry marketing is not made readily available to the public and is therefore difficult to obtain [65–67]. There is also a corresponding paucity of qualitative interview studies that explore marketing techniques involving gambling industry employees [47]. This lack of internal information results in empirical studies primarily taking an interpretative approach with researchers investigating the nature of gambling advertising via content or sentiment analysis. Although these forms of analysis are legitimate methods of elucidating subjective themes and messages within media content, appropriate measures must be taken to ensure trustworthiness [68]. However, amongst such studies in the current review (*n* = 20), only 11 reported the use of numerous coders. Such methodological limitations reduce the reliability of the associated studies and impede the quality of the research area.

The included studies typically included large samples of televised gambling adverts that were representative of those aired to the public. Although the content of televised adverts may be targeted, they are not disseminated based on the personalised data of the audience, thus individuals who watch the same television broadcast will be presented with the same advertisement. In contrast, representative online advertisements may be more challenging to obtain and investigate due to the industry trend of moving away from the use of online blanket marketing and towards the utilisation of individually targeted advertisements that utilise the digitised personal data of the user [47]. Theoretically, individuals could visit the same web page but be presented with different gambling advertisements. Furthermore, although mentioned anecdotally throughout the associated literature, there is a noticeable lack of research that investigates unsolicited pop-up advertising disseminated online and within mobile apps. These advertisements may be difficult to empirically study due (in-part) to their unpredictable and context-specific nature. This review therefore proposes investigation into online gambling advertisements that use personalised data as an additional future research priority in congruence with this popular marketing strategy.

It also appears the gambling advertising sphere may evolve at a speed that the academic literature struggles to keep pace with. The current review indicates that the literature base surrounding the nature and characteristics of gambling advertising has slowly expanded between 2015 and 2020 but remains underdeveloped in terms of scope and methodological diversity. In contrast, much more research has been conducted in relation to the similar areas of tobacco, alcohol and fast-food marketing [69–71]. The majority of available evidence has been conducted in either the UK or Australia. Therefore, alongside the general paucity of existing research, even less information has been produced in relation to other jurisdictions in which gambling and associated advertising have also been liberalised. Without insight into the unique gambling advertising characteristics of jurisdictions other than the UK and Australia, the associated literature remains culturally homogenous. In addition, there is a corresponding paucity of cross-cultural studies that compare the characteristics of gambling marketing based on varying regulatory approaches between jurisdictions. The current review therefore recommends the growth of such studies within the future literature in congruence with the global expansion of the gambling sphere.

### Limitations

The findings of the current review should be considered in light of some potential limitations. Firstly, only studies that were published in the English language were included. Gambling advertising is prevalent across numerous jurisdictions in which English is not the primary language such as Sweden, Spain and France. Therefore, insightful and pertinent studies may have been excluded during the search strategy. Secondly, due to the rapid review methodology utilised, limitations were placed upon the number of databases searched alongside the time dedicated to screening. For example, although the MMAT is a widely used and reputable quality assessment tool [33], it is acknowledged that more in-depth yet time consuming tools are available. Despite these potential limitations, numerous coders were involved in the screening and quality assessment process in order to reinforce the rigor of the current methodology. Furthermore, the protocol for the current review was registered online alongside the inclusion of a search strategy report (Additional File 1) to increase transparency and trustworthiness.

## Conclusions

There is limited research that focuses upon the content, delivery and structural features of emerging gambling advertising. The associated literature base between 2015 and 2020 has slowly expanded but is lacking in volume and diversity. This may be problematic given the findings here suggesting that as digital communication and the liberalisation of gambling advance, so do the intensity and complexity of gambling advertising. Furthermore, the online evolution of gambling advertising has resulted in more interactive adverts in which the promotional intent is less conspicuous than more conventional marketing strategies. There are numerous barriers that hinder empirical investigation into these topics. A deeper understanding and further research into gambling advertising characteristics are therefore warranted in order to effectively minimise potential harm, appropriately regulate gambling advertising and encourage more ethical marketing.

## Supplementary Information

**Additional file 1.** Search Strategy Report. A table denoting the databases used, the search terms and results within the search strategy of the current review

**Additional file 2.** Included Study Summary. Summary of included study characteristics and key findings

## Data Availability

Data sharing is not applicable for this review as the procedure did not involve the construction or analysis of a dataset. Supplementary materials relating to the search strategy and a summary of included studies are provided (Additional files 1 & 2).

## Abbreviations

EGM: Electric gaming machine
MMAT: Mixed-methods appraisal tool
PRSIMA: Preferred Reporting Items for Systematic Reviews and Meta-Analyses
RG: Responsible gambling
T&Cs: Terms and conditions
UK: United Kingdom
