# Supplementary material for: Emergent gambling advertising; a rapid review of marketing content, delivery and structural features
Source: BMC Public Health. 2021 Apr 14;21:718. doi: 10.1186/s12889-021-10805-w (PMC8043759; doi:10.1186/s12889-021-10805-w)
Supplement: Supplementary file 1 — Additional file 1. Search Strategy Report. A table denoting the databases used, the search terms and results within the search strategy of the current review [file 12889_2021_10805_MOESM1_ESM.pdf]

<table>
<tr><th colspan="2">Search strategy: Databases, filters and search terms – conducted 02/06/20</th></tr>
<tr><td>PsychInfo (via proquest) – advanced search</td><td rowspan="2">418 results</td></tr>
<tr><td>Filters:
From 01 January 2015 to 06 June 2020
Scholarly Journals
Empirical Study
Language: English

Terms: ((gambl* OR betting OR casino* OR sports*) AND (market* OR advert* OR promot*) AND (content OR strateg*))</td></tr>
<tr><td>Web of Science (Science Citation Index Expanded & Social Sciences Citation Index) – advanced search</td><td rowspan="2">935 results</td></tr>
<tr><td>Filters:
From 2015-2020
Document type: article
Language: English

Terms: ((gambl* OR betting OR casino* OR sports*) AND (market* OR advert* OR promot*) AND (content OR strateg*))</td></tr>
<tr><td>Google Scholar – free searching</td><td rowspan="2">16 results</td></tr>
<tr><td>Filters:
From 2015-2020
Patents and citations excluded</td></tr>
</table>
