# Supplementary material for: Emergent gambling advertising; a rapid review of marketing content, delivery and structural features
Source: BMC Public Health. 2021 Apr 14;21:718. doi: 10.1186/s12889-021-10805-w (PMC8043759; doi:10.1186/s12889-021-10805-w)
Supplement: Supplementary file 2 — Additional file 2. Included Study Summary. Summary of included study characteristics and key findings [file 12889_2021_10805_MOESM2_ESM.pdf]

Table 1. Summary of included study characteristics and findings.

Study	Authors	Date	Setting	Study Design	Type of advertising investigated	Aims and Objectives	Quality Assessment (MMAT)[a]	Advertising Content Highlighted	Delivery Strategies Highlighted	Mechanics and Structural Features Highlighted
**Abarbanel et al, 2017**	Abarbanel, Gainsbury, King, Hing & Delfabbro	2017	Australia	Qualitative content and textual analysis.	Social casino advertisements (*n* = 115) disseminated via social media.	To understand the compositional elements of social-casino marketing materials and how this messaging is being targeted towards young adults.	*****	1) The use of content and imagery that is likely to appeal to young adults including depictions of young adults and references to pop-culture. 2) The incorporation of themes such as encouragement to participate, the glamorisation and normalisation of gambling. 3) A significant lack of 'responsible gambling' messaging within the advertisements.	Predominantly delivered via Facebook through unsolicited advertisements.	Embedding an 'activity' button within the advertisements that provides the opportunity to directly download the social casino app or accessing the web interface for the game.
**Bestman et al, 2016**	Bestman, Thomas, Randle, Pitt, Daube & Pettigrew	2016	Australia	Mixed-methods interpretative content analysis.	Promotions used on 65 social club websites that advertise both gambling and non-gambling activities.	1) To determine the extent and nature of activities promoted via social club websites. 2) To hypothesise how such promotions may shape attitudes / behaviours.	*****	1) The utilisation of narratives that emphasise better value and increased chances with gambling activities. 2) Endorsing the idea that customers could ease their own financial stress through cash-prizes from gambling. 3) Framing the gambling venue as comfortable and accommodating.	Embedding gambling-related advertisements into the same webpages that advertise family-orientated social club venues.	N/A
**Bradley & James, 2019**	Bradley & James	2019	UK	Mixed-methods involving quantitative (frequency) analysis and sentiment analysis.	The twitter-based marketing activity of 22 UK gambling operators.	To investigate how large gambling companies engage with customers and advertise their products via Twitter.	*****	1) Predominantly using words and narratives that are associated with positive emotions such as trust and joy.	1) Posting between 89 and 202 tweets a day. 2) Posting tweets at 'peak' times such as 11:00, 15:00 and 16:00. 3) Tweeting more on particular days, possibly in relation to current sporting events.	1) Embedding a direct link to the gambling operator/betting page within the advertisement. 2) Utilising unique hashtags that reference sporting events or promote specific offers. 3) Utilising the ability for the operator to engage with customers by replying to their user-requests about bets and odds.
**Bunn et al, 2019**	Bunn, Ireland, Minton, Holman, Philpott & Chambers	2018	UK	Quantitative (frequency) analysis.	Gambling related football shirt sponsorship between 1992 and 2018.	To investigate the prevalence of gambling sponsorship in English and Scottish premier league football.	*****	N/A	1) Significantly increasing the amount of gambling-related shirt sponsorship in English and Scottish Premier League football between the introduction of the UK 2005 Gambling Act and 2017.	N/A
**Critchlow et al, 2020**	Critchlow, Moodie, Stead, Morgan, Newall & Dobbie	2020	UK	Mixed-methods interpretative content analysis.	A range of UK gambling advertisements disseminated via print-press, television, internet websites and email.	To examine the presence and visibility of age-restriction warnings, harm reduction messages and T&Cs within paid-for gambling advertising in the UK.	****	1) The avoidance of including any age restriction warnings, harm reduction messaging or T&Cs in advertisements. 2) When age restriction warnings, harm reduction messages or T&Cs were displayed, the vast majority had very poor visibility due to small font, colour schemes or being positioned outside of the main frame. 3) Specifically, T&Cs contained complex or technical language and stipulations.	N/A	N/A
**Deans et al, 2016**	Deans, Thomas, Daube, Derevensky & Gordon	2016	Australia	Mixed-methods interpretative content analysis.	Sports betting wagering advertisements delivered via television, Youtube and on websites.	To explore and interpret the symbolic appeal strategies used in wagering advertisements that may facilitate the normalisation of gambling.	*****	The incorporation of content that contain appeal strategies aimed primarily at men. These included; thrill and risk, gender stereotypes, peer bonding, patriotism, sexualised imagery, enhancing social status, winning, happiness, power/control and sports fan rituals.	N/A	N/A
**Gainsbury et al, 2016**	Gainsbury, Delfabbro, King & Hing	2016	Australia	Mixed-methods involving quantitative (frequency) analysis and qualitative (thematic) analysis.	Social media marketing utilised by 101 gambling operators.	To comprehensively appraise the extent of and latent messages conveyed within social media being utilised by	****	1) Using social media to promote content relating to products, offers, customer engagement, tips, and features that assist betting. 2) The incorporation of latent messages such	Predominantly advertising gambling products and communicating with customers via Facebook and Twitter. These social media platforms were most	N/A

						the Australian gambling industry.		as glamorisation, emphasising ease of use, encouraging brand engagement (like/follow/comment) and gambling participation. 3) Lack of conspicuous harm reduction messaging within the social media posts.	frequently used by sports-wagering websites.	
**Gainsbury et al, 2015**	Gainsbury, King, Hing & Delfabbro	2015	Australia	Qualitative interviews – analysed thematically.	Gambling marketing and promotion via social media as described by individuals who work for the industry.	1) To explore how gambling operators are using social media to engage with users and promote their products. 2) To understand the considerations and strategies that underpin these actions.	****	1) Interweaving odds related content into 'news' broadcast posts that provide users with sports and betting information. 2) Placing an emphasis on engaging with users in a 'non-commercial' manner to build positive customer relationships. 3) Sharing stories of winning customers. 4) Posting content that encourages brand-engagement (like/follow/comment)	1) Utilising social media platforms for various strategic uses. Facebook primarily used for information and customer feedback; Twitter used for broadcasting news and promotions. 2) Paying for advertising space that is targeted rather than blanket-media marketing.	1) Maximising brand exposure by utilising the 'like' button on social media platforms. 2) Utilising the ability for the operator to engage with customers by replying to their user-requests about bets and odds.
**Håkansson & Widinghoff, 2019**	Håkansson & Widinghoff	2019	Sweden	Mixed-methods involving quantitative descriptive and content analysis.	144 hours of various gambling advertisements (*n* = 891) disseminated via Swedish television.	To describe the extent and content of televised gambling adverts in relation to placement and potential public-health components such as the targeting of specific audiences.	*****	1) Predominantly advertising online casino betting. 2) Incorporating a female-specific theme within the online casino adverts by depicting significantly more female gambling behaviours compared to male gambling behaviours. 2) Un-licensed operators more likely to air content relating to free-bets and offers.	1) Delivering the most amount of televised gambling advertising within the 10pm-2am slot followed by the 6pm-10pm slot (nights and evenings).	N/A
**Hing et al, 2017**	Hing, Sproston, Brook & Brading	2017	Australia	Quantitative (frequency) analysis and descriptive categorisation.	Wagering inducements (*n* = 223) offered across the websites of 30 gambling operators.	To characterise and document the structural features of wagering inducements and analyse their alignment with the goals of 'responsible gambling'.	*****	1) The use of inducement related content comprised of incentivising offers such as; sign-up offers, refer-a-friend offers, refund offers, happy hours, free bets and competitions. 2) Significant lack of 'responsible' gambling messaging within the advertisements.3) T&Cs were almost always displayed outside of the inducement in a separate location on the website. These T&Cs were difficult to find and used complex language.	N/A	1) Bonus bets and offers that have specific play-through requirements before winnings can be withdrawn.
**Houghton et al, 2019**	Houghton, McNeil, Hogg & Moss	2019	UK	Mixed-methods summative content analysis.	The twitter activity (*n* = 8315 tweets) of 5 UK gambling operators and affiliates.	1) To address what content is being posted on Twitter by the UK gambling industry. 2) To provide an understanding of the marketing strategies gamblers encounter on social media.	*****	1) Disseminating content that contains; direct advertising, sports content, humour, updates of current bet status and promotional content. Gambling affiliates more likely to post direct advertising and betting tips. 2) Gambling operators more likely to take an indirect approach using humour to build brand awareness. 3) Significant lack of safer gambling messaging within the tweets.	Gambling affiliates posting around 594 tweets a day on average, with gambling operators posting around 362 tweets.	1) Encouraging user engagement via features such as online polls. 2) Utilising the ability for the operator/affiliate to engage with customers by replying to their user-requests about betting assistance and odds.
**Killick & Griffiths, 2020**	Killick & Griffiths	2020	UK	Mixed-methods content analysis.	The twitter activity (*n* = 3375 tweets) of UK gambling operators during the opening weekend of 2018-2019 premier league football.	1) To examine how gambling operators advertise their products on Twitter. 2) How operators engage with their followers. 3) Implications for the regulation of sports betting advertising.	*****	I) Disseminating promotional content such as user-requested odds, free-bet offers, boosted odds and in-play betting information. 2) Significant lack of 'responsible' gambling messaging within the tweets.	Posting between 33 to 398 tweets a day.	1) Utilising unique hashtags that reference/link to sporting events or promote specific offers. 2) Encouraging user engagement via features such as online polls. 3) Utilising the ability for the operator to engage with customers by replying to their user-requests about bets and odds.
**Lopez-Gonzales, Estévez & Griffiths, 2017**	Lopez-Gonzales, Estévez & Griffiths	2017	UK	Qualitative content analysis using a grounded theory approach.	Televised UK sports betting promotions (*n* = 102) from 2014-2016	To distinguish the themes and narratives that are utilised by gambling operators within sports betting promotions.	****	1) The incorporation of content and narratives within sports betting advertisements that orientate themes of reduced risk. These include 'free money' (offers) and depictions of fun. 2) Content that orientates themes of increased control such as knowledge,	N/A	N/A

								data analysis, masculinity and experience. 3) These themes are framed by depictions of technological features (i.e. mobile gambling use) that are depicted to be control enhancers within the adverts.		
**Lopez-Gonzales et al, 2018a**	Lopez-Gonzales, Estévez, Jiménez-Murcia & Griffiths	2018	Spain / UK	Mixed-methods content analysis.	Televised sports betting advertisements (*n* = 135) that aired in both the UK and Spain between 2014-2016.	To explore how sports betting advertisements present gambling, low nutritional food and alcohol in association with emotionally charged situations and notions of friendship building.	****	1) Content that visually aligns drinking alcohol with sporting culture. 2) Combining depictions of drinking alcohol with emotionally charged situations such as celebrating goals. Friendship bonding linked with alcohol drinking in the context of sports betting.	N/A	N/A
**Lopez-Gonzales, Guerrero-Solé & Griffiths, 2018**	Lopez-Gonzales, Guerrero-Solé & Griffiths	2018	Spain / UK	Qualitative content analysis.	Televised sports betting advertisements (*n* = 135) that aired in both the UK and Spain between 2014-2016.	To understand how advertising normalises betting behaviour by depicting specific behaviours and actions while underrepresenting others.	****	1) Content primarily containing small groups of male characters –however when these characters are shown to be betting, they are often alone. 2) Content that advertises 'in-play' betting by depicting it's use via smart phones and laptops. 3) Depicting characters staking small amounts for large returns (longer odds).	N/A	N/A
**Lopez-Gonzales et al, 2018b**	Lopez-Gonzales, Guerrero-Solé, Estévez & Griffiths	2018	Spain / UK	Qualitative content analysis.	Televised sports betting advertisements (*n* = 133) that aired in both the UK and Spain between 2014-2016.	To examine the structural metaphors that underpin sports betting advertising that may shape the way bettors think about their own betting behaviour and betting in general.	****	Four conceptual metaphors that underpinned the televised adverts for online sports betting were highlighted. 1) aligning the core concept of love for a team with betting on that team. 2) portraying sports betting as a 'market' that is rational, regulated and overseen. 3) Presenting betting as a 'natural' environment. 4) betting is a sport in which bettors are active players.	N/A	N/A
**Newall, 2017**	Newall	2017	UK	Mixed-methods; content analysis and observational/experimental	Televised 'live-odds' advertisements shown during English premier league football between January-February 2016	To investigate the content of 'live-odds' football bets in terms of their complexity and how this may impact bettors' ability to judge the associated probability of such bets.	*****	The incorporation of 'live odds'-related narratives and incentives within the adverts. Specifically, content that is skewed towards advertising more complex bets (containing numerous events) rather than simple bets. Complex bets within the advertisements were seen to have longer odds (and higher potential wins) therefore productive of higher profit margins.	Embedding 'live-odds' gambling advertising before, during or after televised UK Premier League football matches.	N/A
**Newall, 2015**	Newall	2015	UK	Quantitative involving observations of advertisements and analysis of bet probability	UK bookmaker advertisements shown in shop windows (*n* = 179) and on television (*n* = 103) during the 2014 football World Cup.	To distinguish how bookmakers herd with the special bets they offer customers via advertisements and the probability of such bets.	****	The advertisement of 'special' or complex bets (longer odds) rather than simple bets. Such content almost exclusively containing depictions and descriptions of bets with high expected losses and framing sporting events via these bets.	Advertising specific complex bets at a higher frequency via TV and bookmaker shop windows during the 2014 World Cup.	N/A
**Newall et al, 2019**	Newall, Thobani, Walasek & Meyer	2019	UK	Mixed-methods involving quantitative descriptive and content analysis.	Televised 'live-odds' advertisements aired during the 2018 football World Cup.	To measure the extent and explore the key features of 'live-odds' gambling advertising in terms of the sense of "urgency" and 'impulsiveness' represented within them.	*****	The incorporation of 'live odds'-related narratives and incentives within the adverts. Specifically, content that appears to make advertised 'live-odds' bets more impulsive via a qualitative trend skewed towards advertising more complex bets (containing numerous events).	Embedding 'live-odds' gambling advertising before, during or after televised World Cup football matches.	Many 'live-odd' bets could be determined before the football match had ended alongside bets improving in odds (flash-odds) for a limited time only. Suggests such mechanics are designed to create a sense of urgency.
**Newall, Walasek & Ludvig, 2019**	Newall, Walasek & Ludvig	2019	UK	Mixed-methods involving quantitative descriptive and content analysis.	Televised 'request-a-bet' advertisements (*n* = 46) aired during the 2018 football World Cup.	1) To review the content of 'request-a-bet' gambling advertising in relation to UK BCAP regulations. 2) How this	*****	The incorporation of odds-related narratives and incentives within the adverts. Specifically, it is suggested that this content is likely designed to nudge gamblers through multiple	Delivering 'request-a-bet' gambling advertising before, during or after televised World Cup football matches.	Utilising unique hashtags on Twitter such as #getaprice that allow users to create their own bets by requesting odds for

						content frames the illusion of control and overweighted small probability bets.		channels toward bets with larger potential payoffs.		combined events of their choice (longer odds).
**Pitt et al, 2018**	Pitt, Thomas, Bestman, Randle & Daube	2018	Australia	Mixed-methods interpretative content analysis.	Australian gambling advertisements from a range of formats that were disseminated between 2008-2015.	1) To explore the attention strategies utilised within gambling advertising. 2) To inform future research aimed at identifying how such strategies shape the attitudes and behaviours of children.	*****	The incorporation of various attention strategies across advertisements. These included the strategic use of audio, depictions of technology, humour, animations, colour schemes, characters and animals, concepts of winning, social benefits of gambling, celebrities or teams, depicting reduced risk and highlighting sporting success.	N/A	N/A
**Purves et al, 2020**	Purves, Critchlow, Morgan, Stead & Dobbie	2020	UK	Quantitative (frequency) analysis	Gambling marketing in televised broadcasts across a range of professional sports in the UK.	To examine the extent and nature of gambling promotion (verbal and visual) in UK sports following the voluntary ban on such advertising.	*****	1) Visual sponsorship almost always consisted of the gambling brand logo. 2) Significant lack of 'responsible' gambling or harm reduction messaging within the advertisements.	1) Utilising sponsorship rather than commercials to disseminate gambling promotion within boxing, football, rugby, and tennis. Gambling sponsorship appears to be most prevalent in boxing followed by football. 2) Gambling advertising references were usually displayed within the area of play or around the pitch.	N/A
**Rawat, Hing & Russel, 2019**	Rawat, Hing & Russel	2019	Australia	Mixed-methods; ecological momentary assessment and content analysis	Emails and text (*n* = 931) messages sent to customers by gambling operators during sports and racing events	To explore the content of direct messages sent to customers by gambling operators during sporting events.	*****	1) Saturating texts and emails with betting incentives and inducements. Incentives included bonus bets, rewards points, better odds/winnings, and reduced risk. Inducements included bonus or better winnings, refund/stake back offers, and match your stake/deposit. 2) Texts were short and concise while emails were longer and contained more information/graphics	N/A	Embedding a direct link to the gambling operator/betting page within the text/email.
**Stead et al, 2016**	Stead, Dobbie, Angus, Purves, Reith & Macdonald	2016	UK	Qualitative content analysis and interviews	The webpages (*n* = 230) of 10 UK online bingo sites.	1) To identify and analyse in detail the characteristics of online bingo websites. 2) To explain the potential appeal of online bingo in the UK to bingo players	*****	1) Incorporating an easy to navigate, unsophisticated design that is structured to present online bingo as fun, light-hearted, and reassuring. 2) Content and narratives that are aimed at first time users and creating a sense of belonging (tips on 'bingo-lingo', use of 'feminine' colours and inclusive language). 3) Content that encourages users to play bingo on the go.	N/A	N/A
**Thomas et al, 2015**	Thomas, Bestman, Pitt, Deans & Randle	2015	Australia	Mixed-methods interpretative content analysis.	The marketing content of sports wagering operators distributed by across Youtube, Twitter and Facebook.	1) To identify the extent and nature of marketing tactics used by gambling operators on social media. 2) To construct a typology of these tactics to inform future research.	*****	1) Utilising content within marketing strategies that contain humorous videos/memes and language associated with winning. 2) The co-branding of wagering promotions by sporting organisations, codes and athletes. 3) Posting material that often contains information about wagering or sports-related information. 4) Dissemination of incentives and inducements (offers, bonus bets, tips).	1) Merging corporate responsibility initiatives (i.e. cancer awareness) with gambling promotion. 2) Utilising videos on YouTube, Facebook posts and Twitter to promote gambling both directly and indirectly.	1) Utilising unique hashtags on Twitter that reference sporting events or promote specific offers. Similarly, using these hashtags to embed the tweet into existing feeds about sporting events.

Note: [a] MMAT is scored across 5 criteria for each type of study design. https://rb.gy/7qvy3y
